# Butylated hydroxytoluene can protect polyunsaturated fatty acids in dried blood spots from degradation for up to 8 weeks at room temperature

**DOI:** 10.1186/1476-511X-12-22

**Published:** 2013-02-20

**Authors:** Adam H Metherel, Ryan C Hogg, Lindy M Buzikievich, Ken D Stark

**Affiliations:** 1 Department of Kinesiology, University of Waterloo, 200 University Avenue West, N2L 3G1, Waterloo, Ontario, Canada

**Keywords:** Butylated hydroxytoluene, Dried blood spots, High-throughput, Omega-3 biomarker, Peroxidation

## Abstract

**Background:**

Dried blood spots (DBS) from fingertip prick blood can enable high throughput fatty acid profiling but may be prone to lipid peroxidation during storage. The use of butylated hydroxytoluene (BHT) on chromatography paper can prevent polyunsaturated fatty acid (PUFA) loss but examinations on the length of storage times possible are not comprehensive.

**Method:**

In the first study, venous whole blood was saturated on paper strips pre-soaked with 0, 2.5 or 5.0 mg/mL BHT and exposed to air for up to 28 days. In a second study, the effect of sealing DBS on 5.0 mg/mL BHT-soaked chromatography strips in capped test tubes or vacuum sealed polypropylene bags with and without nitrogen purging was examined over eight weeks. The fatty acid composition of the DBS were determined by gas chromatography and the effect of sample storage on omega-3 biomarkers were examined.

**Results:**

PUFA and omega-3 biomarkers in DBS stored without BHT were dramatically reduced by day 3. In general, BHT delayed decreases in eicosapentaenoic + docosahexaenoic acid from baseline (3.2 ± 0.2 wt%) to 28 days (2.6 ± 0.03 wt%) of storage. In the % n-3 highly unsaturated fatty acids (HUFA) in total HUFA biomarker, BHT was more effective at preventing changes, particularly with 5.0 mg/mL BHT where no differences were detected up to 28 days. Sealed storage with BHT tended to increase the stability of the PUFA in DBS and nitrogen purging did not appear to provide additional benefits. The % n-3 HUFA in total HUFA biomarker also appeared to be more stable in the sealed storage study.

**Conclusions:**

The storage of DBS in sealed containers with BHT may prevent PUFA degradation for up to 8 weeks. The % n-3 HUFA in total HUFA biomarker appears to provide a more consistent assessment of omega-3 status throughout storage as compared with other omega-3 blood biomarkers.

## Background

Dried blood spots (DBS) from fingertip prick blood does not require a trained phlebotmist and can enable high-throughput screening of blood omega-3 biomarker status when combined with direct transesterification and fast gas chromatography. Omega-3 blood biomarkers are associated with chronic disease [1-3] and may be useful for health promotion, disease detection and disease prevention strategies in human populations. Previously, DBS analysis has been validated for the determination of fatty acid composition in whole blood [4-7], and has been used to assess fatty acid profiles from elderly individuals [8], young Canadian adults [9], Italian and Tibetan adults [10], and mothers and their newborns [11,12]. Omega-3 blood biomarkers such as the sum of the percentage of eicosapentaenoic acid (EPA, 20:5n-3) and docosahexaenoic acid (DHA, 22:6n-3) (% EPA + DHA) [1] in erythrocytes and the percentage of omega-3 highly unsaturated fatty acids (HUFA, ≥20 carbons, ≥3 carbon-carbon double bonds) in total HUFA (% n-3 HUFA in total HUFA) [3] utilize qualitative fatty acid values in their calculations, and as such DBS analysis remains suitable as blood volumes are often unknown. However, peroxidation of polyunsaturated fatty acids (PUFA) in blood stored on chromatography paper can limit the utility of high-throughput DBS sampling.

Previously, decreases in the PUFA content of erythrocytes are prevented for up to 4 weeks when stored at −20°C [13,14], and for up to 2 years at −80°C [15] in the absence of an antioxidant. However, access to −80°C storage is often limited outside of research institutions. In 1979, it was demonstrated that butylated hydroxytoluene (BHT) improves PUFA recovery in the fatty acid analysis of lipids [16], and it is typically included in routine fatty acid analyses [17]. The addition of BHT to erythrocytes for storage extends PUFA stability from 4 to 17 weeks [13]. Previously, PUFA in DBS with BHT treatment were shown to be stable for between 3 weeks [6] and 3 months [18] at 4°C, and for less than 2 months [18] at room temperature [18]. The aim of the present study is to assess the validity of using different levels of BHT added to chromatography paper to prevent PUFA degradation in DBS samples stored openly at room temperature for one month. In addition, the effect of storing DBS samples with BHT in vacuum sealed polypropylene bags and in capped test tubes with and without nitrogen purging for up to 8 weeks at room temperature on PUFA degradation was examined. This single individual study will provide a basis for the assessment of these storage methods in more extensive storage stability protocols that utilize larger sample sizes.

## Methods

### Study design

All procedures and protocols received prior approval from the Human Research Ethics Committee of the University of Waterloo. In study one, chromatography paper was soaked in three separate concentrations of 2,6-di-*tert*-butyl-4-methylphenol (butylated hydroxytoluene, BHT, Sigma-Aldrich, St. Louis, MO, USA) in methanol. Concentrations of BHT in methanol included 0 mg/mL (control), 2.5 mg/mL and 5 mg/mL [6]. The BHT/methanol mixture was allowed to travel to the top of one sheet of chromatography paper (Whatman Ltd., Sanford, ME, USA) in a thin layer chromatography tank before the paper was removed and air dried. The fatty acid compositions of DBS from each BHT condition were immediately analyzed in triplicate by gas chromatography at time 0. The remaining samples were stored in open test tubes and analyzed in triplicate after 1, 3, 7, 14, 21 and 28 days to track changes in fatty acid composition due to PUFA degradation.

In study two, all strips of chromatography paper were treated with BHT in methanol at a concentration of 5.0 mg/mL. At time 0, DBS were collected in triplicate and immediately transesterified for gas chromatography determination of fatty acid composition. This time 0 determination served as baseline for all four storage conditions, as the sample collection procedures were similar across storage conditions. The remaining samples were randomly assigned to be stored in; a closed screw cap test tube, a closed screw cap test tube following nitrogen purging, a vacuum sealed polypropylene bag sealed with a commercially available vacuum sealer (Foodsaver V2840; Sunbeam, Mississauga, ON), or a vacuum sealed polypropylene bag after nitrogen purging. The fatty acid composition of three samples from each storage condition were determined weekly for eight weeks.

### Blood collection

Blood was obtained by venipuncture from a single fasting male on two occasions corresponding to study one and study two. Venous blood was collected from the antecubital vein into 10 mL evacuated tubes (Vacutainer; Becton Dickinson, Franklin Lakes, NJ) by a trained technician and ethylene diaminetetraacetic acid (EDTA) was added to prevent coagulation. Blood was absorbed onto the thin strips (1 cm × 3 cm) of chromatography paper such that an area of approximately 1 cm^2^ was saturated using a pasteur pipette.

### Fatty acid analysis

Fatty acid composition of each DBS was determined using a method previously described [4]. Fatty acid methyl esters were prepared from DBS by direct transesterification in 14% boron trifluoride in methanol (Pierce Chemicals, Rockford, IL, USA) with hexane using a convectional block heater set at 95°C for 60 minutes. The organic layer containing the fatty acid methyl esters was collected for analysis on a Varian 3900 gas chromatograph (Varian Inc., Mississauga, ON) as previously described [19]. The gas chromatograph was equipped with a DB-FFAP 15 m × 0.10 mm i.d. × 0.10 μm film thickness, nitroterephthalic acid modified, polyethylene glycol, capillary column (J&W Scientific from Agilent Technologies, Mississauga, ON) with hydrogen as the carrier gas. Samples (2 μL) were introduced by a Varian CP-8400 autosampler into the injector heated to 250°C with a split ratio of 200:1. The initial temperature was 150°C with a 0.25 min hold followed by a 35°C/min ramp to 200°C, an 8°C/min ramp to 225°C with a 3.2 min hold and then an 80°C/min ramp up to 245°C with a 15 min hold at the end. The flame ionization detector temperature was 300°C with air and nitrogen make-up gas flow rates of 300 mL/min and 25 mL/min, respectively, and a sampling frequency of 50 Hz.

Fatty acid compositions of all samples are expressed as percent weight of individual fatty acids in total fatty acids. The sum of EPA and DHA (EPA + DHA) and EPA, DHA and n-3 docosapentaenoic acid (DPAn-3) (EPA + DHA + DPA n-3), the percentage of n-3 HUFA in total HUFA, and the ratio of total omega-3 fatty acids to omega-6 fatty acids (n-3/n-6) were calculated to illustrate changes in the omega-3 status in the samples and the differential rates of degradation in the fatty acid subclasses.

### Statistical analysis

Qualitative values for individual fatty acids and fatty acid groups are expressed as mean ± SD. All statistical analyses were performed with the SPSS System (SPSS Inc., Chicago, IL). Various fatty acids and fatty acid groups were examined by one-way ANOVA with individual means compared following a significant F-value by Tukey’s Honestly Significant Different (HSD) post hoc procedure. Significance was inferred at p < 0.05.

## Results

### The effect of different levels of BHT during open air storage at room temperature on fatty acid composition

Total PUFA percentages decreased by 49% without BHT, 15% with 2.5 mg/mL BHT and 6% with 5.0 mg/mL BHT (Table 1). In the HUFA subclass, decreases were 62% without BHT, 34% with 2.5 mg/mL BHT, and 13% with 5.0 mg/mL BHT. These results indicate increasing BHT concentrations provided may reduce the degradation of PUFA and HUFA of DBS. The 5.0 mg/mL BHT condition also appears to protect levels of arachidonic acid (ARA), EPA and DHA in DBS. In contrast, saturated fatty acid (SFA) percentages increased by 30% during storage without BHT and by 10% with 2.5 mg/mL BHT. Monounsaturated fatty acid (MUFA) percentages increased by 24% during storage without BHT and by 9% with 2.5 mg/mL BHT. There was no change in SFA and MUFA percentages with 5.0 mg/mL BHT by day 28 of storage.

**Table 1:** Weight % of fatty acids in dried blood spots at varying BHT concentrations over 28 days

**Name**	**0 d**	**1 d**	**3 d**	**7 d**	**14 d**	**21 d**	**28 d**
	***0 mg/mL BHT***						
**SFAs**	42.1 ± 0.9^a^	40.2 ± 0.5^a^	49.2 ± 0.6^b^	54.1 ± 1.7^c^	56.0 ± 0.3^c^	54.3 ± 0.5^c^	54.9 ± 0.9^c^
**MUFAs**	18.0 ± 0.4^a^	18.7 ± 0.2^a^	22.3 ± 0.3^c^	21.3 ± 0.8^bc^	20.8 ± 0.3^b^	21.8 ± 0.4^bc^	22.3 ± 0.7^c^
**N-6 FAs**	34.0 ± 0.6^a^	35.4 ± 0.1^b^	23.9 ± 0.3^c^	20.1 ± 0.5^d^	18.3 ± 0.1^e^	19.0 ± 0.3^de^	18.2 ± 0.6^e^
**N-3 FAs**	4.17 ± 0.24^a^	4.14 ± 0.03^a^	1.98 ± 0.11^b^	1.45 ± 0.08^c^	1.30 ± 0.13^c^	1.63 ± 0.20^bc^	1.47 ± 0.15^c^
**ARA**	8.07 ± 0.14^a^	8.10 ± 0.17^a^	4.25 ± 0.07^b^	3.20 ± 0.14^c^	2.85 ± 0.07^c^	3.17 ± 0.10^c^	2.92 ± 0.22^c^
**EPA**	0.65 ± 0.10^a^	0.64 ± 0.04^a^	0.29 ± 0.02^b^	0.26 ± 0.03^b^	0.25 ± 0.05^b^	0.25 ± 0.02^b^	0.17 ± 0.02^b^
**DHA**	2.19 ± 0.09^a^	2.18 ± 0.03^a^	0.85 ± 0.05^b^	0.63 ± 0.04^cd^	0.55 ± 0.02^d^	0.73 ± 0.02^cd^	0.77 ± 0.06^cd^
**PUFAs**	38.1 ± 0.8^a^	39.5 ± 0.1^b^	25.9 ± 0.3^c^	21.5 ± 0.5^d^	19.6 ± 0.1^e^	20.6 ± 0.5^de^	19.6 ± 0.4^e^
**HUFAs**	14.1 ± 0.5^a^	14.1 ± 0.2^a^	7.59 ± 0.10^b^	5.60 ± 0.24^cd^	5.02 ± 0.22^d^	5.80 ± 0.08^c^	5.40 ± 0.19^cd^
	***2.5 mg/mL BHT***						
**SFAs**	37.1 ± 0.5^a^	37.1 ± 0.3^a^	37.6 ± 0.5^ab^	36.2 ± 0.7^a^	39.2 ± 0.2^c^	39.0 ± 0.8^bc^	40.9 ± 0.7^d^
**MUFAs**	19.0 ± 0.2^ab^	18.7 ± 0.4^a^	19.1 ± 0.4^ab^	19.8 ± 0.4^c^	19.6 ± 0.1^bc^	20.2 ± 0.2^cd^	20.7 ± 0.1^d^
**N-6 FAs**	36.7 ± 0.3^a^	36.0 ± 0.3^a^	35.9 ± 0.1^a^	36.3 ± 0.4^a^	34.9 ± 0.5^b^	34.8 ± 0.4^b^	32.3 ± 0.3^c^
**N-3 FAs**	4.43 ± 0.10^a^	4.32 ± 0.11^ab^	4.15 ± 0.17^abc^	4.35 ± 0.12^ab^	3.96 ± 0.07^bc^	3.79 ± 0.25^c^	2.84 ± 0.06^d^
**ARA**	9.12 ± 0.07^a^	8.43 ± 0.24^b^	8.28 ± 0.08^b^	8.40 ± 0.12^b^	8.08 ± 0.07^b^	7.56 ± 0.22^c^	5.98 ± 0.13^d^
**EPA**	0.71 ± 0.08^a^	0.73 ± 0.06^a^	0.68 ± 0.06^a^	0.66 ± 0.03^ab^	0.62 ± 0.02^ab^	0.63 ± 0.07^ab^	0.52 ± 0.01^b^
**DHA**	2.40 ± 0.19^a^	2.25 ± 0.10^ab^	2.17 ± 0.06^ab^	2.35 ± 0.06^a^	2.16 ± 0.05^ab^	1.95 ± 0.20^b^	1.42 ± 0.05^c^
**PUFAs**	41.1 ± 0.2^a^	40.3 ± 0.3^ab^	40.0 ± 0.3^b^	40.6 ± 0.4^ab^	38.9 ± 0.5^c^	38.6 ± 0.6^c^	35.1 ± 0.2^d^
**HUFAs**	15.7 ± 0.1^a^	14.8 ± 0.2^bc^	14.4 ± 0.2^bc^	14.9 ± 0.2^b^	14.2 ± 0.1^c^	13.4 ± 0.5^d^	10.4 ± 0.2^e^
	***5 mg/mL BHT***						
**SFAs**	36.6 ± 0.2	36.9 ± 0.4	37.6 ± 0.8	36.0 ± 0.4	37.9 ± 2.6	38.5 ± 0.9	39.0 ± 0.7
**MUFAs**	18.6 ± 0.2^a^	19.0 ± 0.1^a^	19.2 ± 0.2^ab^	19.5 ± 0.3^ab^	21.6 ± 2.4^b^	20.1 ± 0.6^ab^	20.4 ± 0.1^ab^
**N-6 FAs**	36.0 ± 0.5^ab^	36.7 ± 0.1^a^	35.7 ± 0.4^abc^	36.0 ± 0.5^ab^	34.2 ± 0.3^c^	34.8 ± 1.1^bc^	34.2 ± 0.2^c^
**N-3 FAs**	4.59 ± 0.28^a^	4.23 ± 0.07^ab^	4.07 ± 0.02^bc^	4.32 ± 0.08^ab^	3.73 ± 0.26^c^	4.16 ± 0.15^abc^	3.75 ± 0.13^c^
**ARA**	8.68 ± 0.15^a^	8.54 ± 0.19^a^	8.21 ± 0.11^ab^	8.53 ± 0.20^a^	7.60 ± 0.45^b^	8.35 ± 0.12^a^	7.72 ± 0.09^ab^
**EPA**	0.82 ± 0.08^a^	0.71 ± 0.03^ab^	0.62 ± 0.01^b^	0.66 ± 0.04^b^	0.60 ± 0.01^b^	0.62 ± 0.02^b^	0.61 ± 0.04^b^
**DHA**	2.41 ± 0.15^a^	2.28 ± 0.02^a^	2.16 ± 0.03^ab^	2.35 ± 0.03^a^	1.98 ± 0.19^b^	2.25 ± 0.11^ab^	1.96 ± 0.06^b^
**PUFAs**	40.6 ± 0.3^a^	41.0 ± 0.2^a^	39.7 ± 0.4^ab^	40.4 ± 0.6^ab^	37.9 ± 0.6^c^	39.0 ± 1.0^bc^	38.0 ± 0.2^c^
**HUFAs**	15.5 ± 0.2^a^	14.8 ± 0.3^ab^	14.3 ± 0.2^bc^	15.0 ± 0.3^ab^	13.2 ± 0.7^d^	14.8 ± 0.3^ab^	13.5 ± 0.1^cd^

Values are means ± SD, n = 3. BHT, butylated hydroxytoluene; SFA, saturated fatty acid; MUFA, monounsaturated fatty acid; FA, fatty acid; n-6, omega-6; n-3, omega-3; ARA, arachidonic acid; EPA, eicosapentaenoic acid; DHA, docosahexaenoic acid; PUFA, polyunsaturated fatty acid; HUFA, highly unsaturated fatty acid. Values with different superscript letters within a row are significantly different by Tukey’s HSD post hoc procedure (P < 0.05) after a significant F-value by one-way ANOVA (P < 0.05).

**Figure 1:**
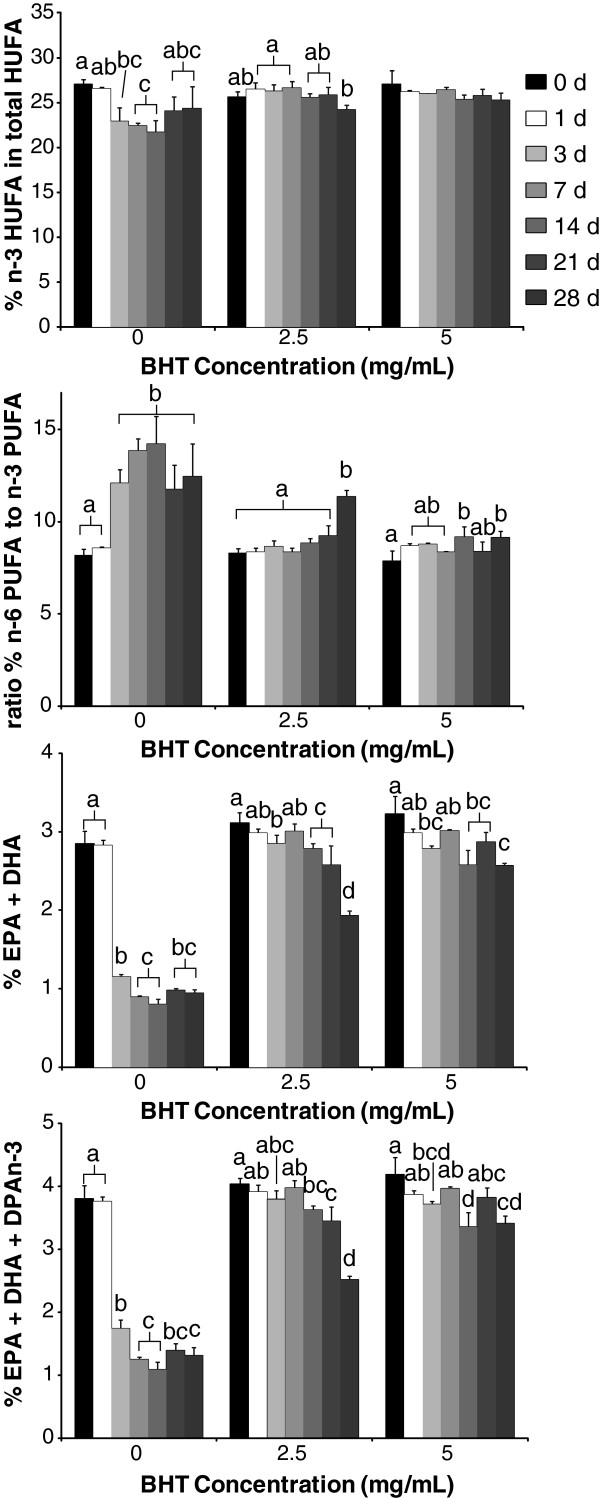
**Omega-3 blood biomarkers in dried blood spot samples stored with 0, 2.5 or 5 mg/mL BHT over 28 days at room temperature. **BHT, butylated hydroxytoluene; HUFA, highly unsaturated fatty acid; PUFA, polyunsaturated fatty acid; EPA, eicosapentaenoic acid; DHA, docosahexaenoic acid; DPAn-3, omega-3 docosapentaenoic acid; n-3, omega-3; n-6, omega-6. Bars represent means with error bars representing the S.D., n = 3. Bars with different letters within BHT condition are significantly different by Tukey’s HSD post hoc procedure (P < 0.05) after a significant F-value by one-way ANOVA (P < 0.05).

Without BHT, blood biomarkers of omega-3 fatty acid status (% n-3 HUFA in total HUFA, EPA + DHA, n-6/n-3 ratio and EPA + DHA + DPAn-3) changed significantly (p < 0.05) after only 3 days of storage (Figure 1). EPA + DHA and EPA + DHA + DPAn-3 levels decreased from baseline (2.84 ± 0.17 and 3.80 ± 0.21, respectively) to day 3 (1.15 ± 0.03 and 1.73 ± 0.14) and remained decreased until day 28 (0.94 ± 0.05 and 1.31 ± 0.12). Conversely, the immediate decrease in the % n-3 HUFA in total HUFA from baseline (27.0 ± 0.6) to day 3 (22.9 ± 1.6) returned to levels not different (p > 0.05) from baseline at day 28 (24.4 ± 2.5). The n-6/n-3 ratio increased from baseline (8.2 ± 0.4) up to day 14 of storage (14.2 ± 1.5) but started to decrease to day 28 (12.5 ± 1.8), although not significantly (p > 0.05).

Storing DBS in the presence of BHT delayed and in certain biomarkers prevented the changes in omega-3 status that are observed when DBS are stored without BHT. With 2.5 mg/mL BHT, baseline % n-3 HUFA in total HUFA (25.7 ± 0.5) was maintained for up to 21 days (25.8 ± 0.9). With 5.0 mg/mL BHT, the % n-3 HUFA in total HUFA did not change from baseline during 28 days. A similar pattern was observed in the ratio of n-6/n-3, but the change from baseline (8.17 ± 0.24) to day 28 (11.4 ± 0.3) was more dramatic in the 2.5 mg/mL BHT condition. There was also a gradual and significant increase in the n-6/n-3 ratio with 5.0 mg/mL BHT. Conversely, EPA + DHA and EPA + DHA + DPAn-3 percentages in the DBS samples were decreased significantly after only 14 days with 2.5 mg/mL BHT and 21 days with 5.0 mg/mL BHT.

### Fatty acid composition and omega-3 biomarkers during sealed storage

**Figure 2:**
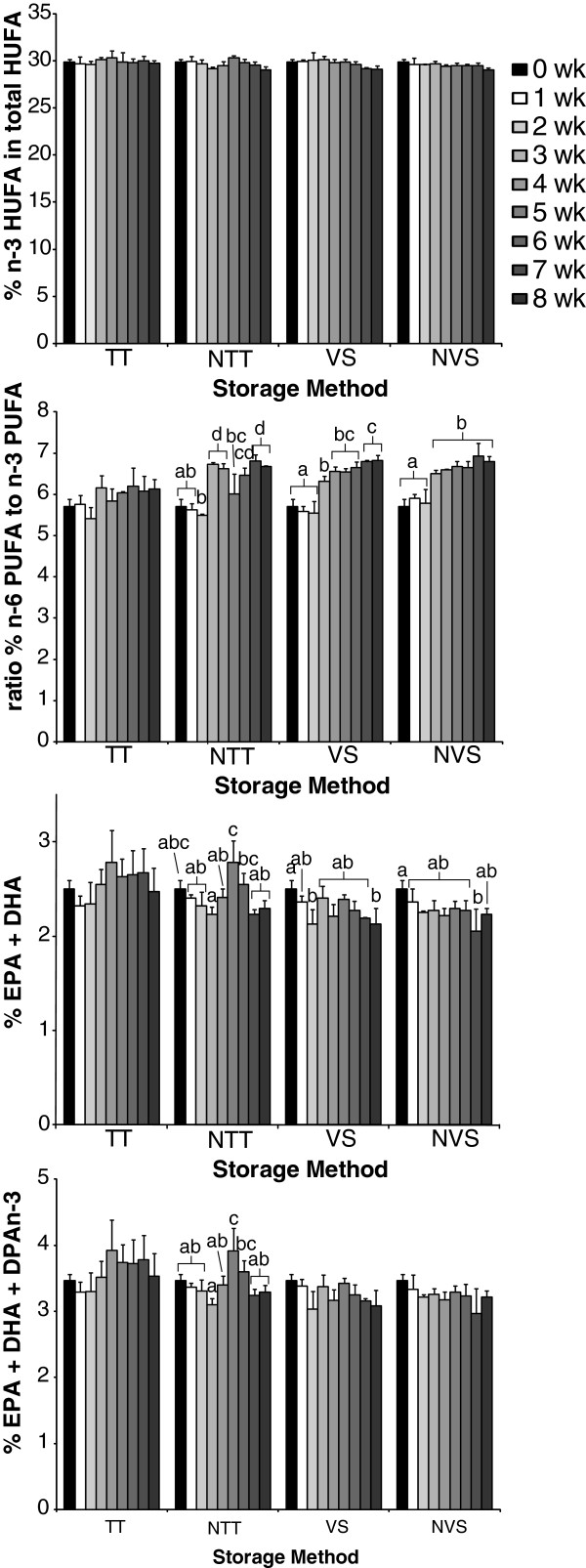
**Omega-3 blood biomarkers in dried blood spot samples after storage for 8 weeks in either a test tube, test tube under nitrogen, vacuum sealed bag or nitrogen dried in vacuum sealed bag. **PUFA, polyunsaturated fatty acid; HUFA, highly unsaturated fatty acid; EPA, eicosapentaenoic acid; DHA, docosahexaenoic acid; DPAn-3, omega-3 docosapentaenoic acid; n-3, omega-3; n-6, omega-6; TT, test tube storage; NTT, test tube storage after nitrogen purging; VS, vacuum sealing in polypropylene bag; NVS, vacuum sealing in polypropylene bag after nitrogen purging. Bars represent means with error bars representing the S.D., n = 3. Bars with different letters within a storage condition are significantly different by Tukey’s HSD post hoc procedure (P < 0.05) after a significant F-value by one-way ANOVA (P < 0.05).

DBS samples stored with 5.0 mg/mL BHT under sealed conditions were examined for up to 8 wks (Table 2). In general, there was a tendency for decreases in the percentages of n-3 PUFA and increases in the percentage of MUFA. These differences were significant in both the vacuum sealed propylene bag conditions and the nitrogen purged sealed test, but surprisingly not in the sealed test tube without nitrogen purging. Changes in the % n-3 HUFA in total HUFA of only 0.4 – 2.8% for all sealed storage conditions throughout the entire study were not significant (p > 0.05) (Figure 2). The ratio of n-6/n-3, and the sums of the percentage of EPA + DHA and EPA + DPAn-3 + DHA demonstrated considerable variation across the weekly measures with significant differences in at least one of the storage conditions.

**Table 2:** Weight % of fatty acids in dried blood spots after BHT treatment and storage by test tube or vacuum sealing

	Week 0*	**Week 1**	**Week 2**	**Week 3**	**Week 4**	**Week 5**	**Week 6**	**Week 7**	**Week 8**
	***Test tube sealed***								
**SFAs**	37.2 ± 0.5	37.6 ± 1.1	38.1 ± 1.4	39.1 ± 0.9	36.4 ± 1.2	39.8 ± 5.2	37.3 ± 0.7	36.7 ± 0.2	41.2 ± 2.3
**MUFAs**	24.6 ± 0.3	25.5 ± 0.5	25.2 ± 0.7	24.1 ± 0.2	25.1 ± 0.8	24.7 ± 2.1	25.6 ± 0.8	25.7 ± 0.3	23.9 ± 1.3
**N-6 FAs**	29.2 ± 0.2	29.7 ± 0.4	29.0 ± 0.4	28.3 ± 0.7	29.3 ± 1.4	29.3 ± 2.8	30.2 ± 0.6	29.8 ± 0.6	28.2 ± 1.7
**N-3 FAs**	5.11 ± 0.12	5.15 ± 0.23	5.36 ± 0.33	4.61 ± 0.33	5.04 ± 0.47	4.86 ± 0.43	4.88 ± 0.32	4.92 ± 0.39	4.60 ± 0.39
**ARA**	6.45 ± 0.04	6.08 ± 0.21	6.12 ± 0.50	6.38 ± 0.46	6.97 ± 0.71	6.87 ± 0.25	6.83 ± 0.54	6.88 ± 0.57	6.51 ± 0.69
**EPA**	0.86 ± 0.06	0.85 ± 0.05	0.80 ± 0.06	0.86 ± 0.05	0.85 ± 0.11	0.85 ± 0.14	0.86 ± 0.04	0.87 ± 0.06	0.81 ± 0.07
**DHA**	1.64 ± 0.04	1.48 ± 0.07	1.55 ± 0.18	1.69 ± 0.11	1.93 ± 0.23	1.78 ± 0.05	1.79 ± 0.22	1.80 ± 0.21	1.66 ± 0.18
**PUFAs**	34.3 ± 0.1	34.8 ± 0.6	34.3 ± 0.7	32.9 ± 1.0	34.4 ± 1.9	34.2 ± 3.2	35.1 ± 0.6	34.7 ± 1.0	32.8 ± 2.0
**HUFAs**	11.6 ± 0.2	11.1 ± 0.4	11.1 ± 0.9	11.7 ± 0.8	12.9 ± 1.3	12.5 ± 0.5	12.5 ± 1.0	12.6 ± 1.0	11.9 ± 1.2
	***Test tube sealed under nitrogen***								
**SFAs**	37.2 ± 0.5	37.3 ± 1.4	35.4 ± 0.3	37.7 ± 1.1	36.0 ± 0.2	36.3 ± 1.1	35.4 ± 0.4	37.1 ± 0.8	36.3 ± 1.4
**MUFAs**	24.6 ± 0.3^a^	25.3 ± 0.5^ab^	26.3 ± 0.4^bcd^	26.0 ± 0.2^bcd^	26.5 ± 0.2^bcd^	25.8 ± 1.2^abc^	26.7 ± 0.2^bcd^	26.9 ± 0.7^cd^	27.4 ± 0.5^d^
**N-6 FAs**	29.2 ± 0.2^ab^	29.7 ± 0.6^abc^	29.9 ± 0.5^abc^	28.7 ± 0.5^a^	30.5 ± 0.4^abc^	30.6 ± 0.7^bc^	31.1 ± 0.4^c^	29.7 ± 0.6^abc^	29.4 ± 1.2^abc^
**N-3 FAs**	5.11 ± 0.12^ab^	5.29 ± 0.04^a^	5.44 ± 0.11^a^	4.27 ± 0.09^d^	4.61 ± 0.14^cd^	5.10 ± 0.30^ab^	4.82 ± 0.18^bc^	4.37 ± 0.12^d^	4.41 ± 0.18^cd^
**ARA**	6.45 ± 0.04^abc^	6.25 ± 0.08^ab^	6.13 ± 0.28^ab^	5.96 ± 0.15^a^	6.37 ± 0.16^ab^	7.04 ± 0.49^c^	6.68 ± 0.19^bc^	6.07 ± 0.16^ab^	6.28 ± 0.21^ab^
**EPA**	0.86 ± 0.06^abc^	0.81 ± 0.02^abc^	0.80 ± 0.06^abc^	0.76 ± 0.04^a^	0.84 ± 0.05^abc^	0.91 ± 0.01^c^	0.89 ± 0.04^bc^	0.78 ± 0.03^a^	0.78 ± 0.05^ab^
**DHA**	1.64 ± 0.04^ab^	1.60 ± 0.04^a^	1.52 ± 0.13^a^	1.47 ± 0.05^a^	1.56 ± 0.04^a^	1.86 ± 0.22^b^	1.66 ± 0.08^ab^	1.46 ± 0.05^a^	1.51 ± 0.05^a^
**PUFAs**	34.3 ± 0.1^abc^	35.0 ± 0.6^abc^	35.3 ± 0.6^bc^	33.0 ± 0.6^a^	35.1 ± 0.5^bc^	35.7 ± 0.4^bc^	35.9 ± 0.6^c^	34.1 ± 0.7^abc^	33.8 ± 1.4^ab^
**HUFAs**	11.6 ± 0.2^ab^	11.2 ± 0.1^ab^	11.2 ± 0.4^ab^	10.6 ± 0.3^a^	11.5 ± 0.3^ab^	12.9 ± 1.0^c^	12.1 ± 0.4^bc^	11.0 ± 0.3^ab^	11.3 ± 0.4^ab^
***Vacuum sealed***									
**SFAs**	37.2 ± 0.5	36.5 ± 0.3	37.6 ± 1.1	37.5 ± 1.7	36.7 ± 0.5	35.3 ± 0.6	35.6 ± 0.5	36.7 ± 0.9	36.9 ± 2.2
**MUFAs**	24.6 ± 0.3^a^	26.3 ± 0.1^b^	26.7 ± 0.5^b^	26.0 ± 0.4^ab^	26.7 ± 1.0^bc^	27.8 ± 0.2^c^	28.1 ± 0.6^c^	27.8 ± 0.6^c^	28.0 ± 0.5^c^
**N-6 FAs**	29.2 ± 0.2	29.6 ± 0.4	28.6 ± 1.2	28.7 ± 0.6	28.2 ± 1.7	30.0 ± 0.1	29.1 ± 0.7	29.1 ± 0.6	28.5 ± 1.7
**N-3 FAs**	5.11 ± 0.12^a^	5.30 ± 0.16^a^	5.17 ± 0.07^a^	4.54 ± 0.19^b^	4.30 ± 0.20^b^	4.59 ± 0.07^b^	4.38 ± 0.20^b^	4.29 ± 0.06^b^	4.18 ± 0.32^b^
**ARA**	6.45 ± 0.04^a^	6.23 ± 0.16^ab^	5.49 ± 0.57^b^	6.15 ± 0.19^ab^	5.84 ± 0.29^ab^	6.28 ± 0.07^ab^	6.04 ± 0.20^ab^	6.00 ± 0.08^ab^	5.87 ± 0.40^ab^
**EPA**	0.86 ± 0.06^a^	0.78 ± 0.02^ab^	0.77 ± 0.05^ab^	0.80 ± 0.04^ab^	0.76 ± 0.06^ab^	0.81 ± 0.02^ab^	0.76 ± 0.04^ab^	0.73 ± 0.01^b^	0.72 ± 0.06^b^
**DHA**	1.64 ± 0.04^a^	1.58 ± 0.05^abc^	1.36 ± 0.14^c^	1.60 ± 0.08^ab^	1.46 ± 0.08^abc^	1.58 ± 0.05^abc^	1.51 ± 0.06^abc^	1.46 ± 0.01^abc^	1.41 ± 0.10^ab^
**PUFAs**	34.3 ± 0.1	34.9 ± 0.5	33.8 ± 1.1	33.3 ± 0.8	32.5 ± 1.9	34.6 ± 0.2	33.5 ± 0.9	33.5 ± 0.6	32.7 ± 2.0
**HUFAs**	11.6 ± 0.2^a^	11.3 ± 0.3^ab^	10.1 ± 1.0^b^	11.2 ± 0.4^ab^	10.6 ± 0.6^ab^	11.5 ± 0.2^ab^	11.0 ± 0.4^ab^	10.8 ± 0.2^ab^	10.6 ± 0.7^ab^
***Nitrogen dried vacuum sealed***									
**SFAs**	37.2 ± 0.5	34.8 ± 0.9	35.9 ± 0.1	37.3 ± 1.2	37.6 ± 1.7	35.7 ± 0.5	36.0 ± 1.5	37.9 ± 3.2	35.4 ± 0.7
**MUFAs**	24.6 ± 0.3^a^	27.2 ± 0.1^bcd^	27.0 ± 0.2^bc^	26.6 ± 0.7^b^	27.0 ± 0.4^bc^	28.1 ± 0.3^de^	27.7 ± 0.5^cd^	28.2 ± 0.3^de^	28.7 ± 0.3^e^
**N-6 FAs**	29.2 ± 0.2	30.1 ± 0.5	29.4 ± 0.1	28.7 ± 0.9	28.4 ± 0.9	29.7 ± 0.2	29.1 ± 0.8	27.7 ± 2.3	29.5 ± 0.3
**N-3 FAs**	5.11 ± 0.12^a^	5.10 ± 0.17^a^	5.10 ± 0.29^a^	4.41 ± 0.10^b^	4.31 ± 0.14^b^	4.46 ± 0.11^ab^	4.38 ± 0.21^b^	4.01 ± 0.50^b^	4.34 ± 0.13^b^
**ARA**	6.45 ± 0.04	6.24 ± 0.22	6.03 ± 0.03	6.02 ± 0.26	5.98 ± 0.19	6.16 ± 0.11	6.06 ± 0.29	5.70 ± 0.54	6.12 ± 0.14
**EPA**	0.86 ± 0.06^a^	0.80 ± 0.04^ab^	0.76 ± 0.02^ab^	0.77 ± 0.02^ab^	0.73 ± 0.04^b^	0.77 ± 0.02^ab^	0.75 ± 0.04^ab^	0.68 ± 0.08^b^	0.75 ± 0.03^ab^
**DHA**	1.64 ± 0.04	1.56 ± 0.10	1.49 ± 0.02	1.50 ± 0.09	1.49 ± 0.04	1.52 ± 0.06	1.52 ± 0.07	1.37 ± 0.16	1.47 ± 0.04
**PUFAs**	34.3 ± 0.1	35.2 ± 0.6	34.5 ± 0.2	33.1 ± 1.0	32.7 ± 1.0	34.2 ± 0.3	33.5 ± 1.0	31.7 ± 2.8	33.8 ± 0.5
**HUFAs**	11.6 ± 0.2	11.3 ± 0.5	10.9 ± 0.1	11.0 ± 0.4	10.8 ± 0.4	11.2 ± 0.2	11.0 ± 0.5	10.1 ± 1.4	11.1 ± 0.3

Values are means ± SD, n = 3. *Week 0 data baseline is the same analysis for all four storage conditions. SFA, saturated fatty acid; MUFA, monounsaturated fatty acid; FA, fatty acid; n-6, omega-6; n-3, omega-3; ARA, arachidonic acid; EPA, eicosapentaenoic acid; DHA, docosahexaenoic acid; PUFA, polyunsaturated fatty acid; HUFA, highly unsaturated fatty acid. Values with different superscript letters within a row are significantly different by Tukey’s HSD post hoc procedure (P < 0.05) after a significant F-value by one-way ANOVA (P < 0.05).

## Discussion

Results of the current study indicate that BHT can provide substantial protection against PUFA degradation in a DBS sample. Total HUFA composition in DBS remains stable in the presence of BHT for up to 21 days in open air although significantly lower levels are observed after 3 and 14 days of storage. Storage stability can be extended to at least 8 weeks when stored and capped in a test tube. These results are not in agreement with a previous study demonstrating that DBS samples wrapped and sealed in foil were unstable during a 2 month storage period [18]. However, the specific BHT concentration used in this previous study is not reported, and as such 5.0 mg/mL of BHT in the present study may explain the improved stability. In addition, our samples were stored in test tubes or vacuum packed polypropylene bags that may provide a more protective seal than foil wrapping.

BHT mediated protection of PUFA is most likely due to free radical scavenging by BHT. The phenol group in BHT is thought to donate a proton to free radicals, thus neutralizing the free radicals and preventing them from accepting hydrogen protons from the methylene groups in PUFA and thereby preventing degradation [20]. The results of the present study support BHT protection against PUFA loss as increasing concentrations of BHT on the chromatography paper demonstrates increased ability to prevent PUFA degradation. Additionally, storing DBS in sealable containers further prevents PUFA loss regardless of storage under an inert gas such as nitrogen.

During open air storage without BHT, omega-3 blood biomarkers responded differently that appear to be dependent on how each biomarker is calculated (Figure 1). Decreases in individual fatty acids from baseline were 23% for 18:2n-6 (data not shown), 33% for 18:3n-3 (data not shown), 47% for ARA, 55% for EPA and 61% for DHA (Table 1) that is in agreement with known kinetic rates of degradation. Omega-3 PUFA have a greater susceptibility for degradation as compared with omega-6 PUFA, as omega-3 PUFA generally contain more double bonds for a given chain length. The kinetic rates of degradation are 1.4 M^-1^ s^-1^ for 18:1n-9, 62 M^-1^ s^-1^ for 18:2n-6, 115 M^-1^ s^-1^ for 18:3n-3, 197 M^-1^ s^-1^ for ARA, 249 M^-1^ s^-1^ for EPA, and 334 M^-1^ s^-1^ for DHA [21,22].

The significant drop in the % n-3 HUFA in total HUFA in DBS after 1 and 3 days in open air without BHT, with a subsequent return to baseline levels beginning at 7 days of storage is intriguing. This may reflect a higher degradation rate of susceptible omega-3 HUFA, with degradation of susceptible n-6 HUFA eventually “catching up” and resulting in a slight and subtle rebound of the % n-3 HUFA in total HUFA. In contrast, the sum of the % of EPA + DHA decreased rapidly and did not return under the same conditions. The % of EPA + DHA is calculated relative to the entire fatty acid pool including large amounts of fatty acids with relatively low degradation rates including palmitic acid (16:0), oleic acid (18:1n-9) and 18:2n-6.

Changes in the n-6/n-3 ratio support the hypothesis of differing degradation rates between omega-3 and omega-6 PUFA. The n-6/n-3 ratio demonstrates an inverse pattern as compared with the % n-3 HUFA in total HUFA with a rapid increase followed by a slight decrease as time passes. The n-6 PUFA with a slower degradation rate is in the numerator while the more rapidly degrading omega-3 PUFA is in the denominator. The differences in the n-6/n-3 ratio and % n-3 HUFA in total HUFA are likely due to the inclusion of 18:2n-6 in the n-6/n-3 ratio. The degradation rate of 18:2n-6 is much slower in comparison with 18:3n-3 and HUFA, and the amount of 18:2n-6 in whole blood is high (23.2 ± 0.4% of total fatty acids) as compared with the other PUFA (38.1 ± 0.8% of total fatty acids) based on baseline values. Although fatty acid peroxidation may still be occurring, the utilization of the % n-3 HUFA in total HUFA blood biomarker may provide a more useful and robust measure of omega-3 status during storage that can mask the effects of HUFA peroxidation and still provide an accurate omega-3 assessment. Our results suggest this may not be the case with the other omega-3 biomarkers assessed.

In the present study, purging with nitrogen did not provide additional protection against PUFA degradation during the 8-week storage period. It is unclear whether nitrogen purging would have provided additional benefits during a longer storage period. Previously, PUFA composition in *O. cincta* (springtail hexapod) has been maintained when saponification and transesterification was performed in nitrogen-filled headspace air [23]. In addition, direct gassing of a dairy beverage enriched with 2% flaxseed oil with nitrogen protected against PUFA degradation [24], and nitrogen has also been used to slow oxidative degradation of perishable food products [25]. In blood, nitrogen prevents fatty acid degradation in phospholipids for at least four weeks in plasma and less than four weeks in erythrocytes during storage at −20°C [14], and in erythrocytes for at least two years when stored at −80°C [15]. Changes in HUFA composition during 8 weeks of nitrogen storage suggest that oxygen exposure may not be the only contributor towards peroxidation in blood samples during storage. In previous studies, erythrocyte exposure to air and pure oxygen *in vitro* for 48 hours at either 37, 26 or 4°C did not result in increased lipid peroxidation as measured by malondialdehyde formation [26]. Plasma bubbled with nitrogen and stored at −20°C also did not prevent PUFA degradation [27]. In healthy cells, approximately 3% of Hemoglobin(Hb)-(Iron)Fe^2+^ is converted to Hb-Fe^3+^ resulting in production of superoxide radicals (·O_2_) [28-30] that can generate lipid peroxyl radicals and lipid hydroperoxides. If the hydroperoxides are not efficiently removed, they can decompose to form more free radicals in the presence of iron that can further exacerbate oxidative damage [31]. Thus, PUFA degradation in the present study may be a result of Fe^2+^-induced peroxidation mechanisms, and BHT has been shown to completely prevent this mechanism of PUFA peroxidation [31-33]. Other potential mechanisms of degradation may include light exposure, thermal degradation and humidity. Although humidity was not controlled for, all samples were stored in the absence of light and at a constant room temperature.

The present study has limitations. Our conclusions are limited to a single individual. A single, homogenous blood sample was used to enable extensive time point characterization of the PUFA degradation and restrict variation to the degradation process. Further assessment of the stability of PUFA in DBS samples need to be conducted on larger cohorts where the influence of other pro- and anti-oxidant blood materials on fatty acid degradation can be addressed. The current study however provides valuable information and timepoints and storage conditions to be examined. An additional limitation is that the baseline PUFA and HUFA levels were higher in study one as compared with baseline values in study two. Although blood samples for study one (different BHT levels) and study two (sealed storage comparisons) were taken from the same individual, they were collected at different time points. As such, comparing results between study one and study two must be made with caution as the potential for PUFA degradation may have been increased in study one. Total omega-3 PUFA measures of approximately 4% in study one and 5% in study two are higher than average DBS values determined in young Canadian adults [9], but similar to values determined in an elderly Canadian population [8]. The higher omega-3 fatty acid composition of blood with the potential for increased peroxidation in the present study may therefore underestimate the PUFA stability of DBS samples from the general North American population.

## Conclusions

Pretreatment of chromatography paper with BHT for DBS sampling may protect against PUFA degradation in a concentration dependent manner for up to 21 days at room temperature, and subsequent sealing of DBS in test tubes or vacuumed polypropylene bags can further protect against PUFA degradation for a minimum of 8 weeks. The % n-3 HUFA in total HUFA biomarker appears more resistant to changes due to PUFA degradation given it is a calculation based on fatty acids with relatively similar peroxidation rates. The % n-3 HUFA in total HUFA may have the potential to provide more accurate determinations of omega-3 fatty acid status following extended DBS storage. Future studies on the mechanisms of PUFA degradation during DBS storage to determine the main determinant of oxidation are required. Improving storage methods for DBS samples to allow for storage at room temperature would be beneficial for population screening for omega-3 biomarker determinations of disease risk and nutritional intake, and in field studies, particularly in developing countries and communities where clinical resources may be limited.

## Abbreviations

ARA: Arachidonic acid;BHT: Butylated hydroxytoluene;DBS: Dried blood spot;DHA: Docosahexaeonic acid;EPA: Eicosapentaenoic acid;HUFA: Highly unsaturated fatty acid;MUFA: Monounsaturated fatty acid;NTT: Nitrogen test tube;NVS: Nitrogen vacuum sealed;PUFA: Polyunsaturated fatty acid;SFA: Saturated fatty acid;TT: Test tube;VS: Vacuum sealed

## Competing interests

The authors declare that they have no competing interests.

## Authors’ contributions

AHM drafted the manuscript and was involved in study design and data analysis. RCH carried out sample collection and storage and participated in sample preparation and analysis. LMB carried out sample collection and storage and participated in sample preparation and analysis. KDS conceived of the study and participated in its design and coordination and helped to draft the manuscript. All authors read and approved the final manuscript.
